# A role for dopamine in the peripheral sensory processing of a gastropod mollusc

**DOI:** 10.1371/journal.pone.0208891

**Published:** 2018-12-26

**Authors:** Jeffrey W. Brown, Brittany M. Schaub, Bennett L. Klusas, Andrew X. Tran, Alexander J. Duman, Samantha J. Haney, Abigail C. Boris, Megan P. Flanagan, Nadia Delgado, Grace Torres, Solymar Rolón-Martínez, Lee O. Vaasjo, Mark W. Miller, Rhanor Gillette

**Affiliations:** 1 Program in Biophysics and Computational Biology, University of Illinois, Urbana, Illinois, United States of America; 2 College of Medicine, University of Illinois at Urbana-Champaign, Urbana, Illinois, United States of America; 3 School of Molecular and Cellular Biology, University of Illinois at Urbana-Champaign, Urbana, Illinois, United States of America; 4 School of Integrative Biology, University of Illinois at Urbana-Champaign, Urbana, Illinois, United States of America; 5 Institute of Neurobiology and Department of Anatomy & Neurobiology, University of Puerto Rico, Medical Sciences Campus, San Juan, Puerto Rico, United States of America; 6 Department of Molecular & Integrative Physiology and the Neuroscience Program, University of Illinois, Urbana, Illinois, United States of America; Nanjing University, CHINA

## Abstract

Histological evidence points to the presence of dopamine (DA) in the cephalic sensory organs of multiple gastropod molluscs, suggesting a possible sensory role for the neurotransmitter. We investigated the sensory function of DA in the nudipleuran *Pleurobranchaea californica*, in which the central neural correlates of sensation and foraging behavior have been well characterized. Tyrosine hydroxylase-like immunoreactivity (THli), a signature of the dopamine synthetic pathway, was similar to that found in two other opisthobranchs and two pulmonates previously studied: 1) relatively few (<100) THli neuronal somata were observed in the central ganglia, with those observed found in locations similar to those documented in the other snails but varying in number, and 2) the vast majority of THli somata were located in the peripheral nervous system, were associated with ciliated, putative primary sensory cells, and were highly concentrated in chemotactile sensory organs, giving rise to afferent axons projecting to the central nervous system. We extended these findings by observing that applying a selective D_2_/D_3_ receptor antagonist to the chemo- and mechanosensory oral veil-tentacle complex of behaving animals significantly delayed feeding behavior in response to an appetitive stimulus. A D_1_ blocker had no effect. Recordings of the two major cephalic sensory nerves, the tentacle and large oral veil nerves, in a deganglionated head preparation revealed a decrease of stimulus-evoked activity in the former nerve following application of the same D_2_/D_3_ antagonist. Broadly, our results implicate DA in sensation and engender speculation regarding the foraging-based decisions the neurotransmitter may serve in the nervous system of *Pleurobranchaea* and, by extension, other gastropods.

## Introduction

Dopamine (DA) is a neurotransmitter found pervasively throughout the animal kingdom. It serves common functions in motor control, sensory gating, and reward systems in such disparate clades as arthropods, molluscs, nematodes, and vertebrates [1–6].

Histochemical and immunohistological studies have shown that the central ganglia of gastropod molluscs contain relatively few catecholaminergic cell bodies [7–11], with dopamine being the only catecholamine found in significant quantities in gastropods [12–13]. Despite these sparse numbers, DA plays important roles in motor pattern selection and regulation in opisthobranchs and their sister clade, the pulmonates [14–18]. Notably, specific identified dopaminergic (DAergic) neurons in the buccal ganglia of the opisthobranch *Aplysia* and the pulmonate *Helisoma* can drive the feeding central pattern generator (CPG) when stimulated [16,17,19,20]. A giant DAergic neuron also serves as an integral element of the respiratory pattern generators in the pedal and visceral ganglia of *Lymnaea* and *Biomphalaria* [11,21,22].

However, quite notable are the findings of considerably more DAergic elements in the peripheral nervous system (PNS) of several gastropods [8,11,23–26]. The peripheral localization and proposed sensory functions of DA in the soft-bodied gastropods are quite unusual relative to its central localization and functions in the skeletonized arthropods, annelids, and vertebrates. We were prompted to investigate the peripheral sensory-motor network of the predatory sea-slug *Pleurobranchaea californica* based on the observations that the predator learns to avoid specific prey odor associated with noxious prey defenses [27] and that considerable sensory computations are being carried out before transmission of chemotactile information to the central nervous system (CNS) [28]. We have documented, compared, and extended the findings of DA localization in gastropods to *Pleurobranchaea* by localization of tyrosine hydroxylase-like immunoreactivity (THli), based on the rate-limiting enzyme in the DA biosynthetic pathway.

Additionally, we found that sulpiride, a selective antagonist of mammalian D_2_/D_3_ receptors that blocks dopaminergic synapses in gastropod molluscs [20,29,30], significantly delayed biting in response to food stimuli presented to hungry specimens when the chemical was externally applied to the chemotactile oral veil-tentacle complex (OVTC). Similar application of the selective D_1_ antagonist SCH-23390, which also exhibits pharmacoactivity in gastropods [31,32], did not significantly alter the time to initiate biting at food. We employed analogous pharmacological manipulation of the OVTC using sulpiride in an electrophysiological paradigm, recording from two major cephalic sensory nerves, the tentacle and large oral veil nerves (TN/LOVN), in a deganglionated head preparation. We observed that sulpiride significantly attenuated evoked neural responses in the TN immediately following treatment, suggesting that the increased latency to bite in the behavioral task might owe to a reduction in sensory information reaching the CNS. Collectively, these results support a role for DA in PNS processing of sensory information in gastropods and invite further investigation of whether DA’s documented role as a neurochemical substrate of food-driven reward and decision in vertebrates and other invertebrate phyla might extend to gastropod molluscs (e.g., [4,5,33,34]).

## Materials and methods

### Immunohistochemistry

Eight specimens of *Pleurobranchaea californica* (100–400 g) were obtained from Monterey Abalone Company (Monterey, CA). They were anesthetized with an injection of 330 mM MgCl_2_ (30–50% body volume), and viscera were removed through a midline incision running the length of the mantle. Ganglia and peripheral tissues were dissected out and pinned to a Sylgard-lined Petri dish in saline of the following composition: 460 mM NaCl, 10 mM KCl, 55 mM MgCl_2_, 11 mM CaCl_2_, and 10 mM HEPES, buffered to pH 8.0. Samples were then fixed for 1 hour in a chilled 4% paraformaldehyde solution containing 27% sucrose. Fixed tissues were washed (5 times, 20 min., room temperature) in 80 mM phosphate buffer containing 2% Triton X-100 and 0.1% NaN_3_ (PTA solution).

Following preincubation with normal goat serum (0.8%), tissues were immersed (48 h, room temperature) in the primary antibody. Catecholaminergic neurons were detected with a mouse monoclonal antibody (DiaSorin, Stillwater MN; Product No. 22941) generated against rat tyrosine hydroxylase (lot LNC1 purified from rat pheochromocytoma PC12 cells). Primary antibody dilutions ranged from 1:300 to 1:100 in PTA (see [11,35,36]).

Following primary antibody incubation, ganglia and tissue samples were washed repeatedly in PTA (5 times, 30 min, room temperature) and incubated in secondary antibodies conjugated to fluorescent markers (Alexa 488 goat anti-mouse IgG (H+L) conjugate or Alexa 546 goat anti-mouse IgG (H+L); Molecular Probes). Secondary antibody dilutions ranged from 1:1,000 to 1:600 in PTA. Due to the large size of some samples, incubation times ranged from two to several weeks before labeled neurons could be optimally visualized.

Processed preparations were initially examined on a Nikon Eclipse fluorescence microscope. Selected samples were then imaged on a Zeiss Pascal Laser Scanning Confocal Microscope (Carl Zeiss Microscopy, LLC, Thornwood, NY). Stacks of optical sections (0.2–1.5 μm) were collected to generate maximum-intensity projections. Confocal images were captured in the Zeiss LSM 5 Image Browser (Version 3.1.0.11).

### Behavior

Animals were tested (N = 6 for D_1_ antagonist; N = 24 for D_2_/D_3_ antagonist) in 1-3-gallon plastic aquariums filled with ASW (Instant Ocean, Blacksburg, VA) and maintained under temperature control (11–14° C).

Readiness-to-feed was measured as previously described [37]. Ascending tenfold dilutions of trimethylglycine betaine (Sigma-Aldrich, St. Louis, MO) from 10^−6^ to 10^−1^ M were applied across the OVTC of the animals with a Pasteur pipette in 1.5 mL volumes over a period 10 s, with two-minute intervals between each application. Betaine readily elicits feeding behavior and is a pervasive osmolyte secreted by *Pleurobranchaea*’s invertebrate prey [37]. A two-part feeding threshold was recorded for each animal, given as the concentrations of betaine that elicited 1) proboscis extension and 2) biting. Only specimens that bit at betaine were judged to be sufficiently hungry for use in the experiment.

**Fig 1:**
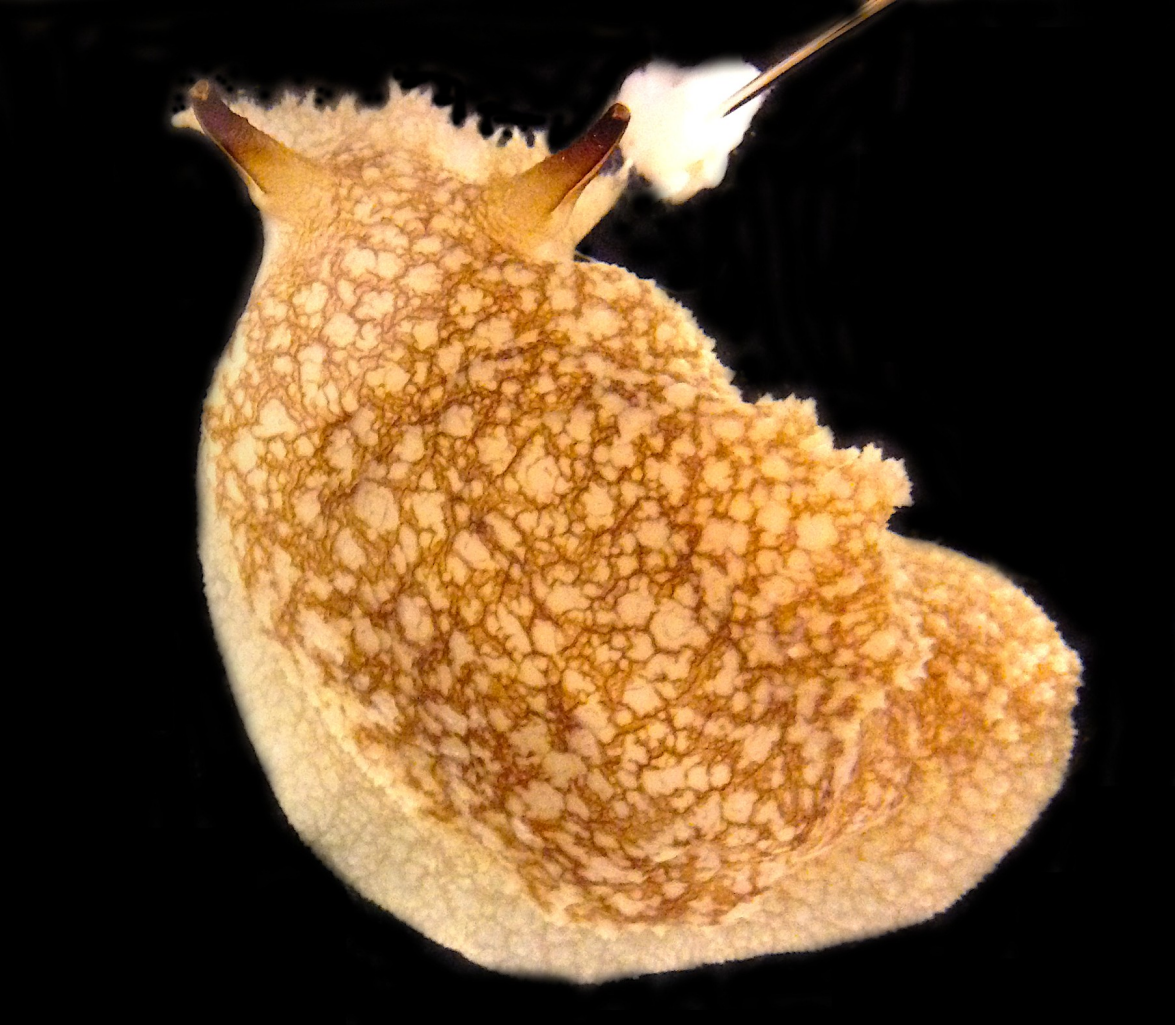
A specimen of *Pleurobranchaea californica* engaged in the food-localization task.

A piece of raw shrimp cut to a standard size was impaled by a rigid metal skewer and gently placed on one of the animal’s tentacles, randomly selected by coin-flip. The shrimp was held stationary, allowing the specimen to move its OVTC along the stimulus until biting was initiated (Fig 1); the latency between shrimp placement and the initiation of biting was recorded. The contralateral tentacle was similarly tested 10 minutes after the cessation of prior biting behavior. Animals were prevented from consuming shrimp during the experiment.

To apply DA antagonists, specimens were subsequently raised gently above the water to expose the OVTC. Solutions of either the D_1_ antagonist SCH-23390 (R(+)-7-Chloro-8-hydroxy-3-methyl-1-phenyl-2,3,4,5-tetrahydro-1H-3-benzazepine hydrochloride; Sigma-Aldrich) or the D_2_/D_3_ antagonist sulpiride (Sigma-Aldrich) were applied to the dorsal surface of a randomly selected side of the OVTC with a fine paintbrush, while the other side was painted similarly using ASW as a control. This procedure typically lasted thirty seconds, after which the animal was lowered back into the aquarium. Both reagents were dissolved in ASW at 100 μM and buffered at a pH of 8.0; a small quantity of dimethyl sulfoxide (DMSO; < 5% of the total solvent volume) was used in initially dissolving the sulpiride.

The shrimp application protocol was repeated 5 minutes following sulpiride treatment on both the control and experimental tentacles. As before, applications on each tentacle were conducted 10 minutes apart and in a random order. Post-treatment bilateral shrimp exposure was conducted only once with each specimen to minimize handling effects. For the same reason, food-seeking data following the washout of the DA antagonist could not be reliably collected, and such trials were therefore not pursued. Control experiments (N = 6) in which pure DMSO was applied unilaterally to the OVTC were employed to evaluate whether DMSO itself affected latency to bite.

### Electrophysiology

Eighteen specimens were anesthetized through cooling to 4° C. The head and intact CNS were dissected away from the body and pinned to a Sylgard-lined dish. Tentacle and large oral veil nerves on one side were cut proximal to the cerebropleural ganglion. The preparation was then submerged in a 6-L Plexiglas flow chamber and perfused with a constant flow of ASW (~50 mL/minute) at 12±1° C.

The distal ends of the TN and LOVN were recorded with suction electrodes. The electrodes were connected via a differential AC amplifier (Model 1700, A-M Systems, Sequim, WA) to a data acquisition system (PowerLab 8/30, ADInstruments, Dunedin, New Zealand). Records were digitized and recorded in LabChart 7.3 (ADInstruments) at a sampling rate of 10 kHz.

Stimulation before and after sulpiride treatment along the oral veil and tentacles was performed with glass Pasteur pipettes with fire-polished tips (3–5 mm diameter). Pipettes were positioned 1–2 cm in front of the targeted OVTC locus and then gradually extended to contact and gently rub the target site for 2 s. Interstimulus intervals were 30 s. Stimuli were applied at either of two loci along the OVTC, corresponding to the most sensitive regions for the two nerves: for the LOVN, this was 5–10 mm ipsilateral to the OVTC midline, and for the TN, the ipsilateral tentacle [28]. 3–6 stimulations at each site were employed before and at 5 and 60 minutes after sulpiride application.

Sulpiride was applied in either of two manners: 1) immersion of the preparation in a bath of 100 μM sulpiride (N = 11) or 2) swabbing one side of the submerged OVTC with a paintbrush dipped in 100 μM sulpiride (N = 7). Pilot data indicated that sulpiride was maximally effective in attenuating stimulus responses in the TN and LOVN 5–16 minutes following treatment. Accordingly, 5 minutes elapsed between sulpiride treatment and the first set of post-treatment stimulation trials. In the majority of cases, a second set of post-treatment trials were conducted 60 minutes following sulpiride application to assess washout effects; where this set of measurements could not be obtained, it was due to the loss of the nerve recording during washout.

### Data analysis

Statistics were computed using both InStat 3.10 (GraphPad, La Jolla, CA) and SAS Studio 3.71 (SAS Institute Inc., Cary, NC). In electrophysiological experiments, nerve recordings were analyzed with homebrew scripts written in MATLAB R2013a (MathWorks, Natick, MA). The total number of extracellular potentials (spikes) exceeding a specific amplitude threshold (±5–10 μV, depending on the integrity of the nerve-electrode seal) was tabulated over every 2-s OVTC stimulation. Spikes counted during the 2 s prior to every stimulation were then subtracted from the spikes tallied during the 2-s stimulation, in an effort to isolate stimulus-evoked from spontaneous nerve activity. Finally, for each preparation, these adjusted spike tabulations were averaged across replications in each testing epoch (pre-treatment, 5 min. post-treatment, and 60 min. post-treatment). Photoshop CC 2015.1 (Adobe) was utilized to adjust the color parameters and background of Fig 1. Stacks, z-series, overlays, and calibrations were generated using ImageJ software (v. 1.43u, NIH public domain). Images were imported to CorelDRAW 10 (Corel, Ottawa, Canada) or Microsoft PowerPoint (v. 14.0.0, Redmond, WA) files for addition of labels, adjustment of brightness/contrast, and organization of panels.

Parametric tests were used to determine statistical significance, with Shapiro-Wilk tests utilized to assess normality and Levene’s tests used to confirm homogeneity of variance. In many instances (noted in the Results section), sets of measurements were log-transformed in order to render data normal and/or homoscedastic for statistical analysis; in such cases, statistics deriving from specific models (e.g., *F* and *p* values) are reported based on transformed data, while the means and standard errors of the raw (untransformed) data are presented. For behavioral data, two-way repeated measures ANOVAs were employed to judge mean differences in latencies to bite, both before and after OVTC painting and between the control and experimental sides of the OVTC, with simple main effects analysis performed to reveal specific pairwise differences where appropriate. A paired t-test was used to evaluate the effects of applying pure DMSO to one side of the OVTC. Differences in mean evoked nerve responses before, at 5 min. following, and at 60 minutes following sulpiride applications across electrophysiological experiments were analyzed with a weighted-means one-way repeated measures ANOVA with Tukey’s post hoc tests; each nerve response mean across all preparations (three means per preparation, corresponding to each of the three testing periods) was weighted with the inverse of its associated standard error (i.e., 1/SEM), such that data from preparations exhibiting greater variability across repeated measurements received proportionally less weight in the ANOVA. Aggregate means and SEMs (i.e., reflecting averaging across all experiments) reported in the Results section were also weighted in this manner. All reported *p* values are two-tailed.

## Results

Results of THli on *Pleurobranchaea* are presented together with comparisons with observations from other heterobranch gastropods.

### Cerebropleural ganglion

**Fig 2:**
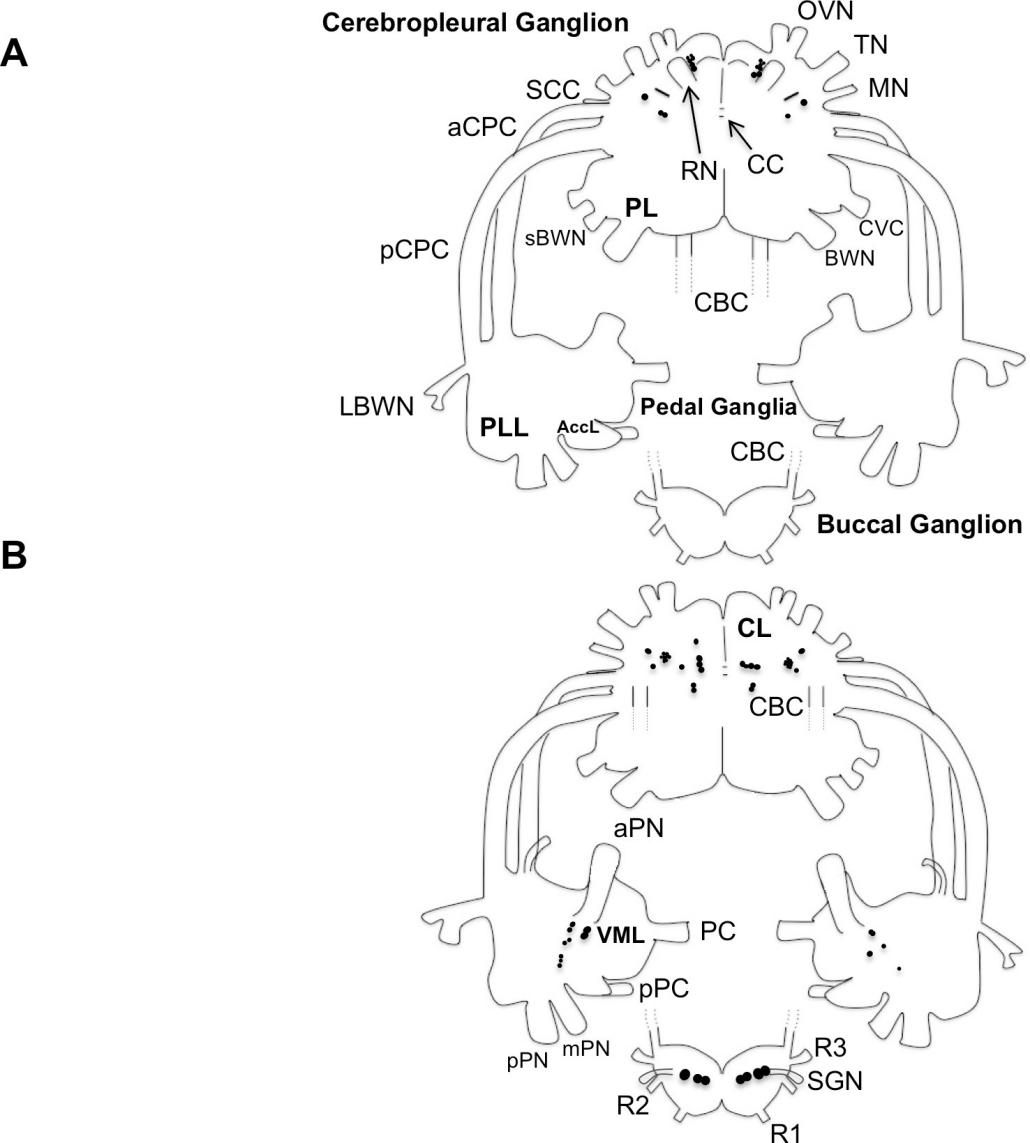
Summary diagram of the distribution of tyrosine hydroxylase-like immunoreactive neurons in the central nervous system. Somata are indicated as black circles on the dorsal (A) and ventral (B) surfaces of the cerebropleural, pedal, and buccal ganglia (modified from [38]; cells not drawn to scale). The cerebrobuccal connectives (CBC) are shown cut here for clarity. Cerebropleural ganglion abbreviations: aCPC, anterior cerebropedal connective; BWN, body wall nerve; CBC, cerebrobuccal connective; CC, cerebral commissure; CL, cerebral lobe; CVC, cerebrovisceral connective; MN, mouth nerve; OVN, oral veil nerve; PL, pleural lobe; pCPC, posterior cerebropedal connective; RN, rhinophore nerve; sBWN, small body wall nerve; SCC, subcerebral commissure; TN, tentacle nerve. Pedal ganglia abbreviations: AccL, accessory lobe; LBWN, lateral body wall nerve; pPC, parapedal commissure; PC, pedal commissure; PLL, posterior lateral lobe; pPN, posterior pedal nerve; VML, ventromedial lobe. Buccal ganglion abbreviations: R1-R3, buccal roots 1–3; SGN, stomatogastric nerve.

**Fig 3:**
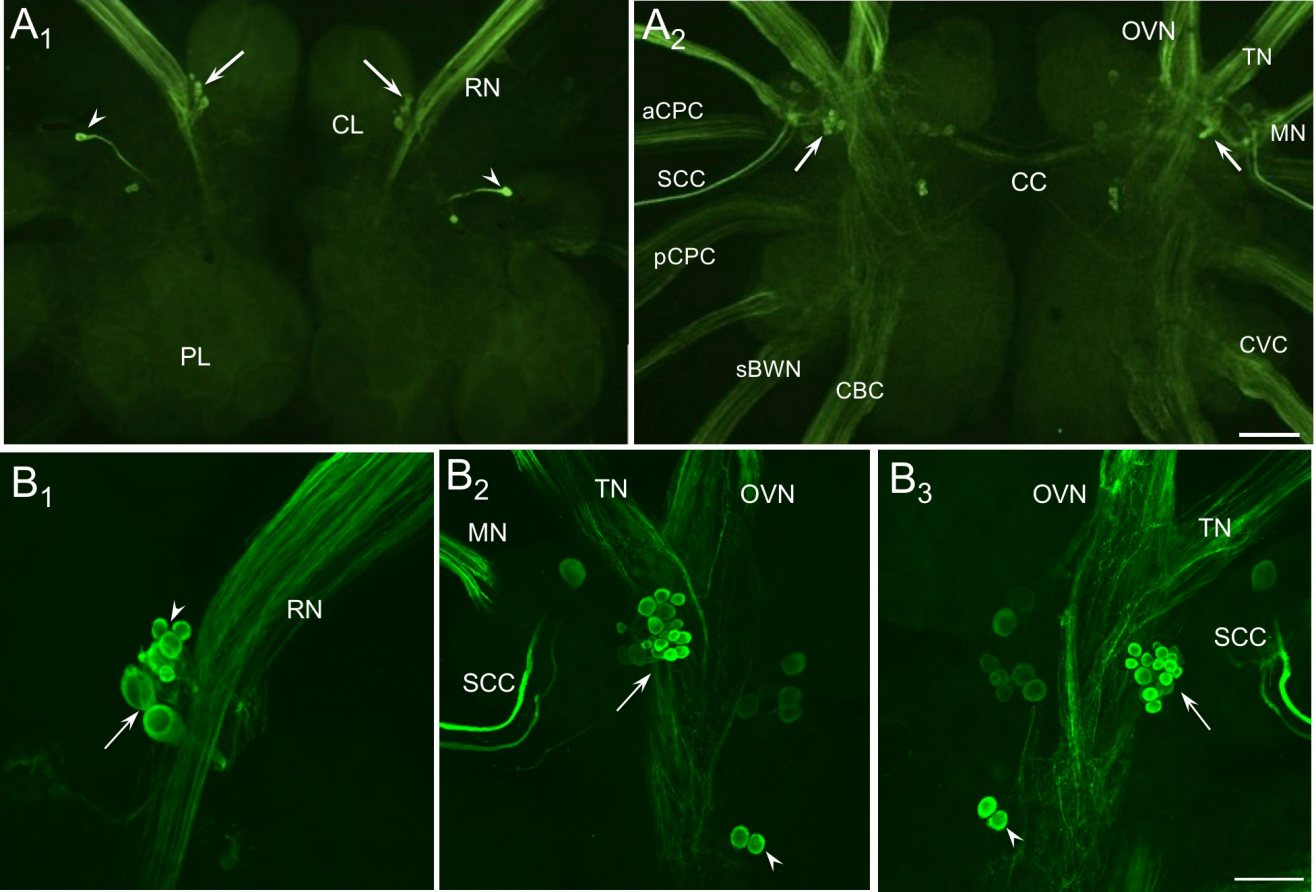
Tyrosine hydroxylase-like immunoreactivity (THli) in the cerebropleural ganglion. (A_1_) Dorsal surface: THli fibers were abundant in the rhinophore nerve (RN). A cluster of 6–8 small cerebral neurons were stained proximal to the origin of each RN (arrows). An additional pair of cells was present lateral to each RN, one of which (arrowheads) projected a prominent fiber toward the midline. THli neurons were not observed in the pleural lobe. (A_2_) Ventral surface: the majority of THli neurons in the cerebral ganglion were located near the confluence of the oral veil (OVN), tentacle (TN), and mouth nerves (MN). THli fibers were abundant in each of these nerves. One cluster of 15–20 small (10–20 μm) neurons was located near the origin of each mouth nerve (arrows). Calibration bar = 400 μm, applies to A_1_ and A_2_. (B_1_) Dorsolateral quadrant of the right cerebral ganglion. At higher magnification, the cluster of THli neurons situated at the origin of the RN was observed to be composed of heterogeneous cell bodies, with larger (30–50 μm) somata located more posterior (arrow) and smaller (10–30 μm) cells in a more anterior position (arrowhead). (B_2_, B_3_) Ventrolateral quadrant of the right and left cerebral ganglia, respectively. The most intensely labeled cells were present in the cluster at the base of the MN (arrows) and exhibited less diversity in size, staining intensity, and segregation than the dorsal cluster cells. Bilateral homologs in this cluster were highly symmetrical with respect to number, location, and intensity. A pair of small (15–20 μm) neurons was located posteromedial to the confluence of the TN and OVN (arrowheads), while a less brightly staining cluster of 3–5 somata lay anterior to those (asterisks). Calibration bar = 100 μm, applies to b_1_-b_3_. Abbreviations: aCPC, anterior cerebropedal connective; BWN, body wall nerve; CL, cerebral lobe; CVC, cerebrovisceral connective; PL, pleural lobe; pCPC, posterior cerebropedal connective; sBWN, small body wall nerve; SCC, subcerebral commissure.

Approximately 25–30 small to medium-sized (10–50 μm) somata within the cerebral lobes of each cerebropleural hemiganglion exhibited THli (Figs 2 and 3). Immunoreactive cell bodies were located near both the dorsal and ventral surfaces, and their distribution was bilaterally symmetric except where noted. The pleural lobes of the ganglion lacked immunolabeled neurons.

On the dorsal surface of the cerebropleural ganglion, a cluster of a 6–8 THli somata was located medial and tangential to the origin of rhinophore nerve (RN; Fig 3A_1_, arrows, and 3B_1_). This population was heterogeneously sized, with larger (30–50 μm) somata located more posteriorly (Fig 3B_1_, arrow) and smaller (10–30 μm) cells in a more anterior position (Fig 3B_1_, arrowhead). The remaining dorsal THli cell bodies were located posterolateral to the origin of the RN. One or two somata were positioned just lateral to the previously characterized A-cluster [39], though their axons were not readily discernable. Anterolateral to these cells was a strongly fluorescent soma (Fig 3A_1_ and 3B_1_ arrowhead) whose axon remained visible along a posteromedial trajectory for approximately 400–600 μm before receding ventrally toward the cerebral commissure.

Neurons stained near the RN in *Pleurobranchaea* were present in both *Aplysia* and *Phestilla*, though the latter species exhibited only two such somata [9,10]. Conserved among all three species were the 2–4 posterolateral immunoreactive somata, which in *Aplysia* and *Phestilla* sent axons crossing the cerebral commissure and 1–3 others posteromedial to the former. Two small THli somata located at the base of the posterior tentacular nerve in *Aplysia* and the homologous RN in *Phestilla* were absent in *Pleurobranchaea*. *Pleurobranchaea* further lacked 1–2 larger THli somata at the origin of the anterior cerebropedal connective that were located at the base of the homologous nerve in *Aplysia* [9,10].

On the ventral surface of the cerebropleural ganglion, THli somata were located near the confluence of the oral veil, tentacle, and mouth nerves (OVN, TN, and MN; Fig 3A_2_ and 3B_2_ and 3B_3_); note that the LOVN is the larger of two major divisions of the OVN, which bifurcates immediately after emerging from the cerebropleural ganglion (Fig 2). A cluster of 15–20 small (10–20 μm) intensely immmunoreactive cells was located immediately posterior to the convergence of the OVN and TN (Fig 3A_2_, 3B_2_ and 3B_3_, arrows). More posteriorly, two brightly stained ventral somata (15–20 μm) were positioned slightly medial to the origin of the TN (Fig 3B_2_ and 3B_3_, arrowheads). In addition to these strongly staining cells, a group of somewhat larger (25–30 μm), 3–5 less bright somata were present medial to the confluence of the OVN, TN, and MN (Fig 3B_2_ and 3B_3_), and a single cell was located more laterally near the origin of the MN and the subcerebral commissure.

Two THli fiber tracts were present in the cerebropleural ganglion commissure (Fig 3A_2_). A larger, anterior tract ran through the cerebral commissure while a smaller group of fibers crossed more posteriorly in the presumed pleural commissure. While THli fibers were resolved in every cerebropleural nerve except the small dorsally located optic nerves, the most conspicuously stained fibers were observed in the OVN, TN, and MN, which carry sensorimotor information to and from the cephalic region [40].

Clear homologies on the ventral side of *Pleurobranchaea*’s cerebropleural ganglion were difficult to establish, but clusters of small, putatively catecholaminergic somata at the confluence of the OVN, TN, and MN were similarly present in *Phestilla* [10] and *Aplysia* [9]. Several homologous THli cells, including those stained at the base of *Aplysia*’s cerebropedal connective, one large cell at the base of the cerebrobuccal connective, and CBI-1, a neuron projecting into the cerebrobuccal connective that is known to modulate feeding [41], were not obvious in *Pleurobranchaea*, although the differing topography in *Pleurobranchaea*’s cerebropleural ganglion leaves open the possibility that homologous neurons could be in different positions. As with *Aplysia* and *Phestilla*, THli cells were absent in the pleural lobes of *Pleurobranchaea*.

### Pedal ganglia

**Fig 4:**
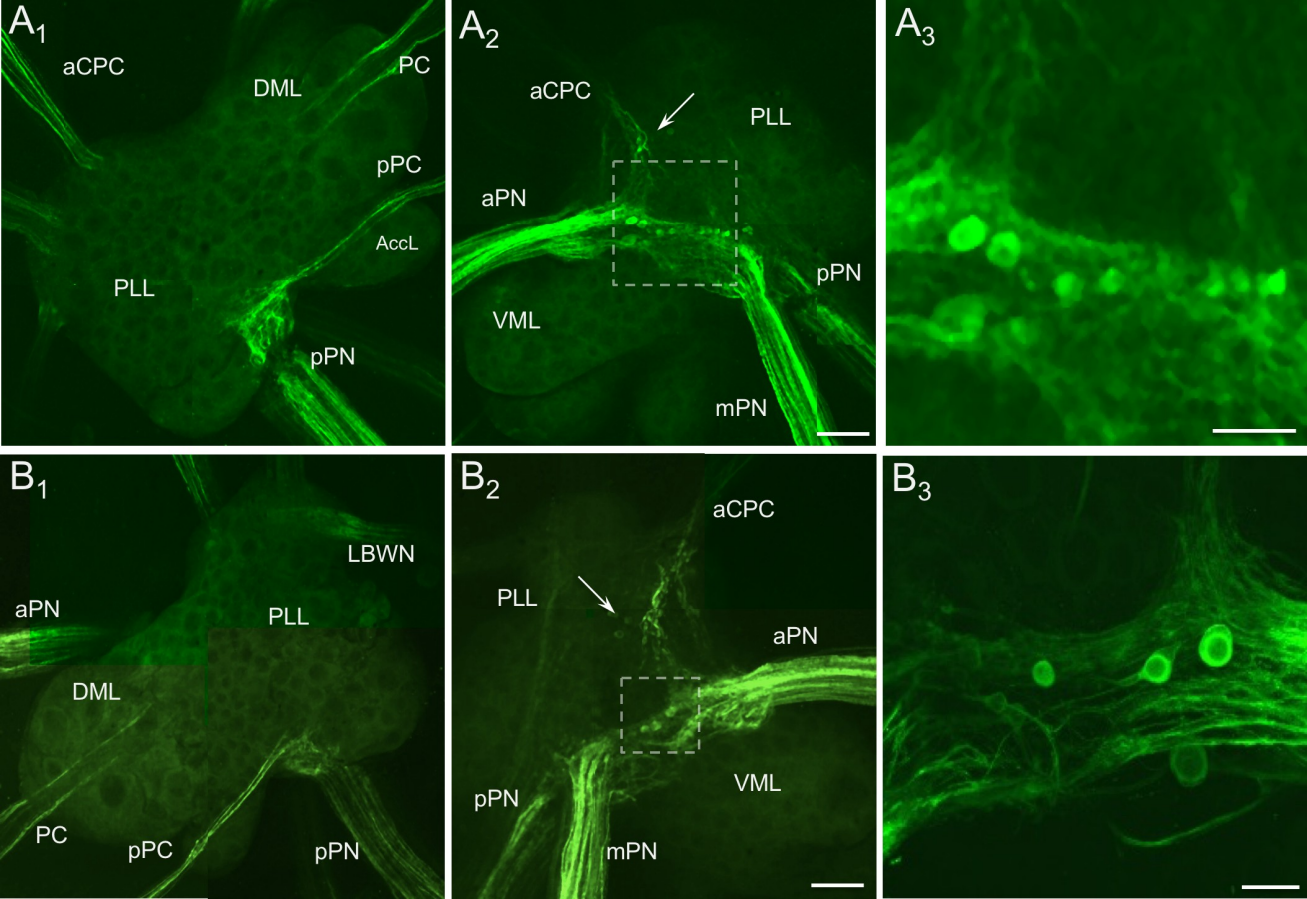
THli in the pedal ganglia. (A_1_, B_1_) Dorsal surfaces of the left and right pedal ganglia, respectively. Immunoreactive fibers were present in each of the nerves and connectives, but no THli cell bodies were observed. (A_2_, B_2_) Ventral surfaces of left and right pedal ganglia, respectively. THli fibers were abundant in the anterior and medial pedal nerves (aPN, mPN). Labeling was also observed in 8–10 and 4–6 small (10–30 μm) neurons at the confluence of the aPN and mPN in the left and right ganglia, respectively. Several additional small THli neurons (10–15 μm) were situated just lateral to the anterior cerebropleural connective (aCPC; arrows). Calibration bar = 200 μm, applies to A_1_, A_2_, B_1_, and B_2_. (A_3_, B_3_) Higher magnification of the regions enclosed by dashed boxes in A_2_ and B_2_, respectively. The neurons embedded in the fiber tract exhibited diverse sizes and staining intensities. Calibration bar for A_3_ = 100 μm; calibration bar for B_3_ = 50 μm. Abbreviations: AccL, accessory lobe; DML, dorsomedial lobe; LBWN, lateral body wall nerve; pPC, parapedal commissure; PC, pedal commissure; PPL, posterior lateral lobe; pPN, posterior pedal nerve; VML, ventromedial lobe.

No THli cell bodies were observed on the dorsal surface of the pedal ganglia (Fig 4A_1_ and 4B_1_). Two distinct fiber tracts were present in the pedal commissure (Fig 4A_1_ and 4B_1_), and a tract of fibers ran between the posterior pedal nerve (pPN) and the parapedal commissure. On the ventral surface of each pedal ganglion (Fig 4B_2_ and 4B_2_), major fiber tracts coursed between the anterior pedal nerve (aPN) and the medial pedal nerve (mPN). Six to ten small (10–30 μm) immunoreactive cells were embedded within this fiber tract at the medial edge of the ventromedial lobe (Figs 2B, 4A_2_, 4B_2_, 4A_3_, and 4B_3_). These neurons were heterogeneous in both size and staining intensity, and were similar to putative homologs in *Aplysia* [9]. A second ventral cluster of 4–6 small (10–15 μm) lightly stained THli somata was located anterolateral to the first group, lateral to the origin of the anterior cerebropedal connective (Fig 4A_2_ and 4B_2_, arrows). Immunoreactive axons were observed in all pedal nerves, with the aPN and mPN containing the most THli fibers.

### Buccal and stomatogastric ganglia

**Fig 5:**
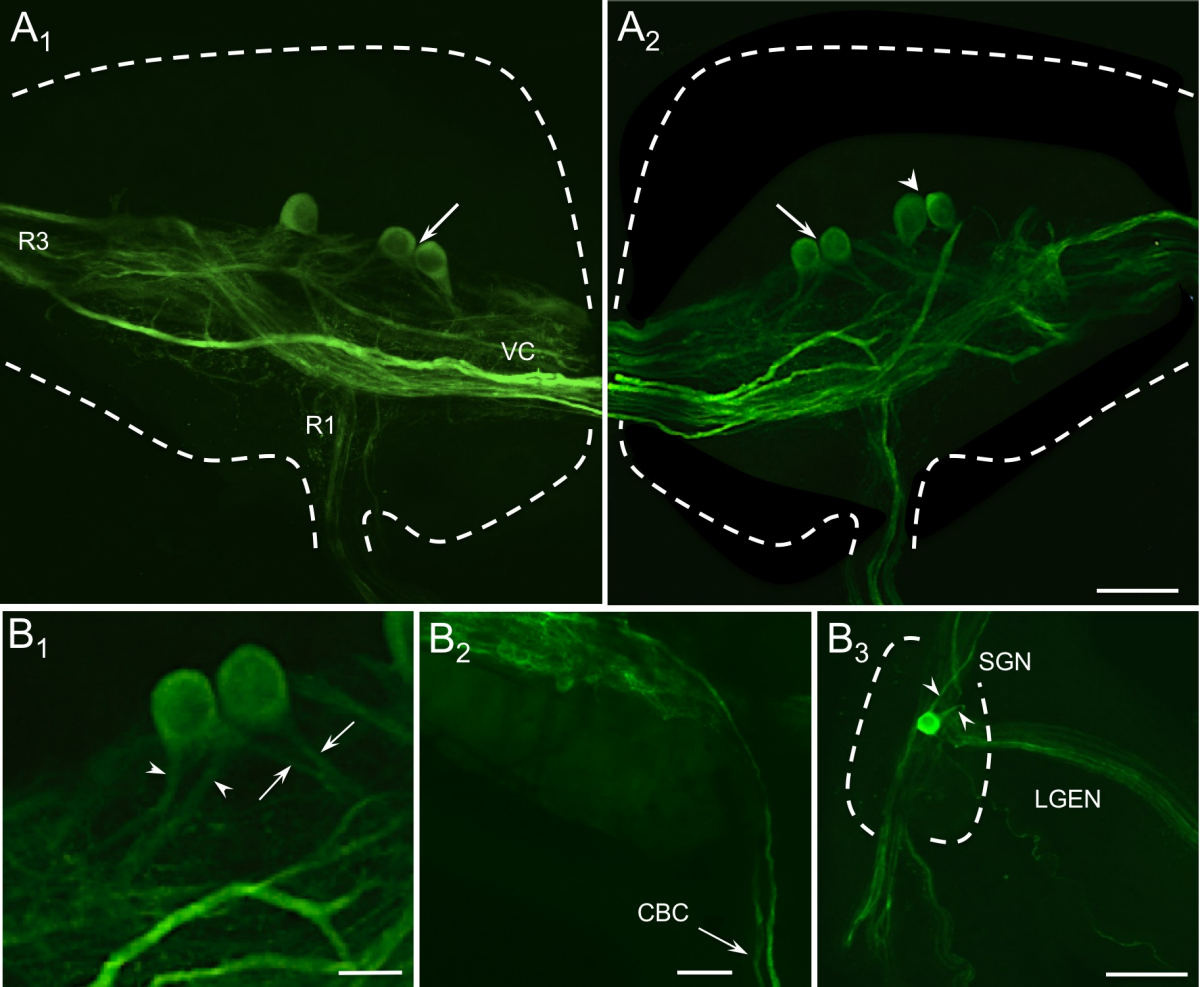
THli in the buccal nervous system. (A_1_) Ventral view of the right buccal hemiganglion. Outline of the ganglion is marked by the dashed blue line. THli fibers coursed through the ventral commissure (VC) and through the buccal neuropil. The majority crossed the ganglion toward buccal root (R3) but several fibers projected into the first buccal root (R1). Three neurons were stained on the anterior margin of the immunoreactive fiber tract projecting into R3. The two medial neurons were contiguous (arrow). (A_2_) Ventral view of the left buccal hemiganglion. Four neurons were present in the medial region of the ganglion, including a medial pair (arrow) and a lateral pair (arrowhead). There was no immunoreactivity on the dorsal surface of the ganglion. Calibration bar = 100 μm, applies to A_1_ and A_2_. (B_1_) Higher magnification of the medial pair of THli cells in the left buccal hemiganglion. Both cells had two fibers originating from the soma, one of which projected medially (arrows) while the other projected laterally (arrowheads). Calibration bar = 40 μm. (B_2_) Anterolateral region of left buccal hemiganglion, ventral view. Two stout axons were present in the cerebrobuccal connective (CBC). Calibration bar = 100 μm. (B_3_) A single THli neuron was located in the stomatogastric ganglion (STG), which is demarcated by the dashed blue line. Two fibers originated from the cell soma (arrowheads), one projecting toward the periphery via the lateral gastroesophegeal nerve (LGEN) and the other toward the CNS via the stomatogastric nerve (SGN). Calibration bar = 100 μm.

Seven medium-sized (35–40 μm) THli somata were present on the ventral surface of the buccal ganglion (Figs 2B, 5A_1_ and 5A_2_). Three neurons present in the right hemiganglion consisted of a medial pair (Fig 5A_1_, arrow) and single lateral neuron, while the left hemiganglion contained four cells segregated into a medial (arrow) and a lateral (arrowhead) pair. These ventral cell bodies were positioned along the anterior margin of the buccal neuropil, into which they projected fibers. Two stout axons could be discerned emanating from several of the buccal THli cell bodies (Fig 5B_1_). Similar bipolar neurons, designated B20, have been shown to drive feeding network motor output in *Aplysia* [16–19].

While fine THli fibers predominated in the buccal nerves projecting to the periphery, each of the cerebrobuccal connectives (CBCs) contained only two large-caliber axons. Within the stomatogastric ganglion, a single immunoreactive soma was stained (Fig 5B_3_). This cell body projected fibers into two adjacent nerves, the stomatogastric nerve (projecting toward the buccal ganglion) and the lateral gastroesophageal nerve, which arises from the stomatogastric nerve as it bifurcates at the ganglion [42]. Additional THli fibers were located in these nerves, as well as in the medial gastroesophageal nerve.

### Visceral ganglion

**Fig 6:**
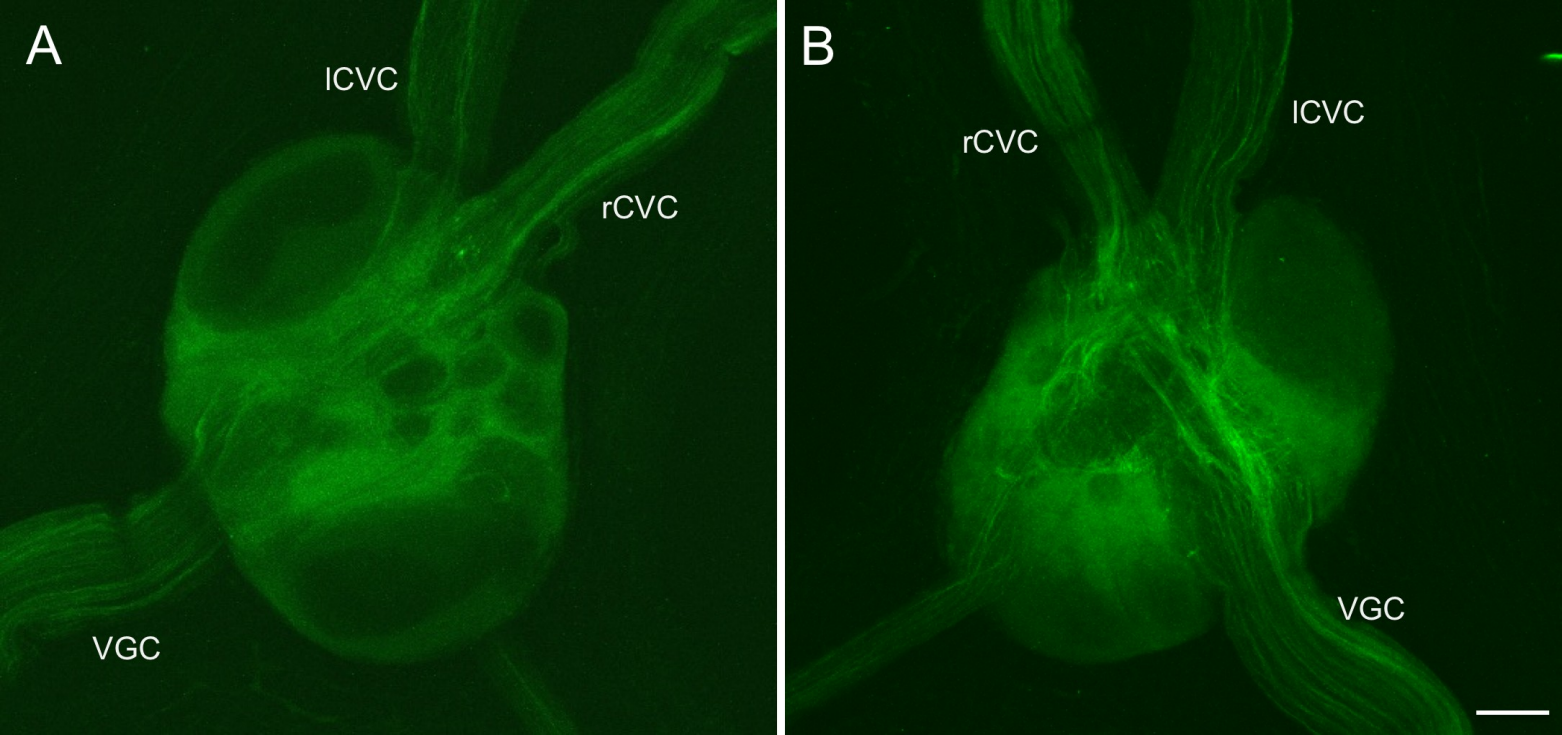
THli in the visceral ganglion. (A) Dorsal view of the visceral ganglion. No immunoreactive somata were observed. (B) Ventral view of the visceral ganglion. THli fibers with an anteroposterior orientation were found passing through the ventral surface of the ganglion, colocated between one of two cerebrovisceral connectives (lCVC, rCVC) and the visceralgenital connective (VGC). Calibration bar = 80 μm, applies to A and B.

No THli neuron somata were present in the visceral ganglion of *Pleurobranchaea* (Fig 6). However, numerous THli fibers coursed through the ganglion, including those in the right and left cerebrovisceral connectives that link the visceral ganglion to the remainder of the CNS. Some of these fibers, which could be more clearly viewed from the ventral aspect of the ganglion (Fig 6B), contributed to fascicles that passed completely through the ganglion to the nerves projecting to the periphery, such as the visceralgenital connective. Some THli axons gave rise to collaterals within visceral ganglion neuropil.

### Tentacle ganglia and nerves

**Fig 7:**
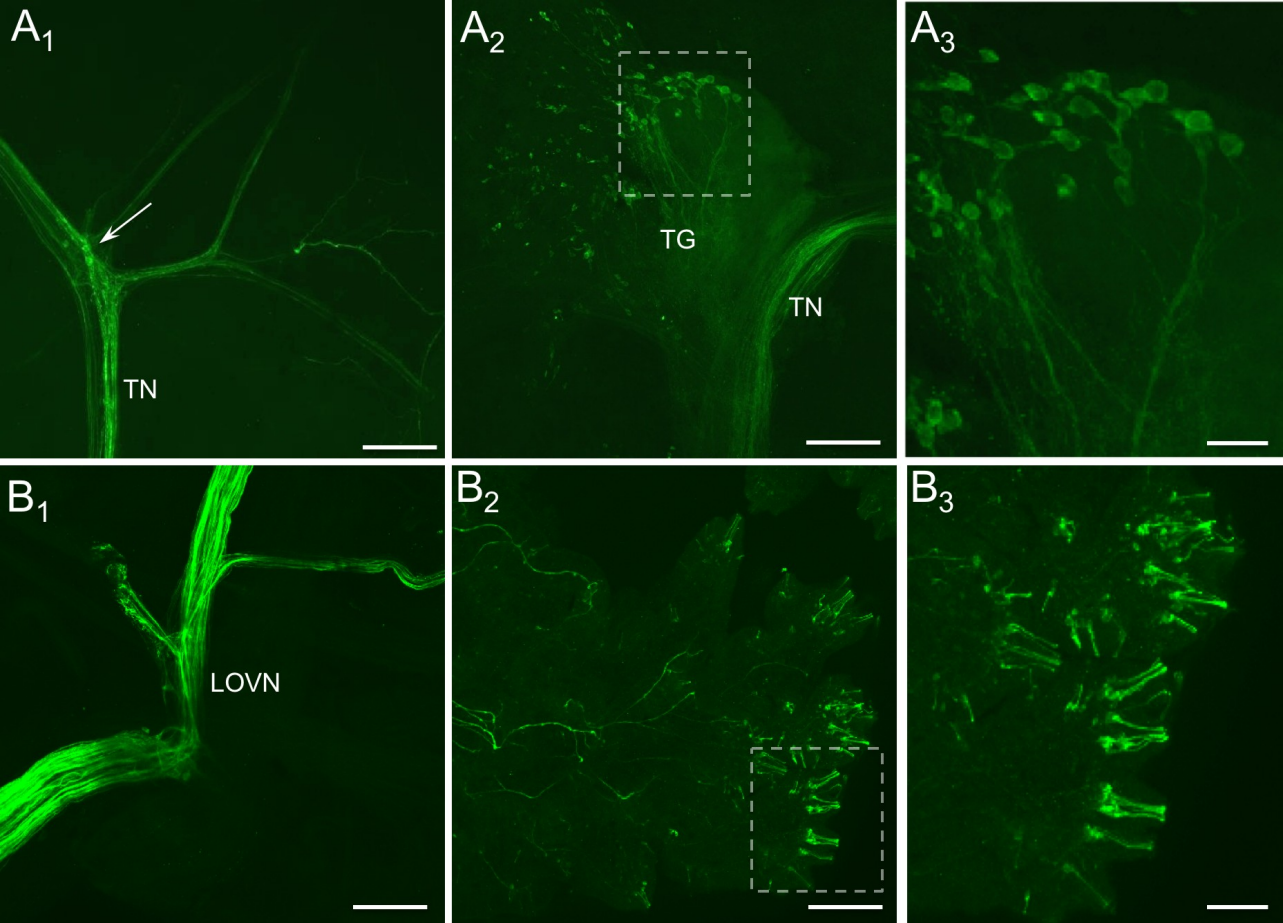
THli in peripheral cephalic tissues. (A_1_) Immunoreactive fibers were present in each of the tentacle nerve (TN) branches. Two to three small (15–20 μm) neurons were resolved at one of the bifurcations (arrow). Calibration bar = 200 μm. (A_2_) Small (5–20 μm) neurons were present in the most distal region of the tentacle ganglion (TG). Calibration bar = 100 μm. (A_3_) The largest somata in the TG gave rise to fibers that projected toward the CNS via the TN. Calibration bar = 30 μm. (B_1_) Numerous immunoreactive fibers were present in the peripheral branches of the lateral oral veil nerve (LOVN). (B_2_) THli fibers branched repeatedly throughout the oral veil epithelium, sometimes terminating in papillae. Calibration bar = 100 μm. (B_3_) Higher magnification of the area enclosed by the dashed white rectangle in panel B_2_. Groups of specialized, cilia-like terminations within the papillae projecting from small somata (< 5 μm) penetrated the oral veil epithelium. Calibration bar = 30 μm.

Consistent with observations of the proximal tentacle nerve (Fig 3A_2_ and 3B_3_), portions of the nerve located in the periphery exhibited a dense concentration of THli fibers (Fig 7A_1_). Immunoreactive fibers were similarly observed in the various medially, dorsally, and ventrally projecting branches arising from the main process of the TN within the head, and were present in the three nerve roots distal to the tentacle ganglion. Small THli neurons were frequently observed at branch points of the TN (Fig 7A_1_, arrow).

The tentacle ganglia are the largest peripheral ganglia found in *Pleurobranchaea* [43]. They are located near the base of the tentacles, approximately 5–10 mm posteromedial to the tips. THli was observed in dozens of small (5–20 μm), brightly stained somata distributed along the outer layer of the tentacle ganglia (Fig 7A_2_). These cell bodies were located distal to the junction of the TN with the ganglion. Many of the larger THli somata projected fibers in the direction of the TN, forming a diffuse network of neuropil in the core of the ganglion (Fig 7A_3_).

### Oral veil

Portions of the oral veil are innervated by the TN, OVN, and MN [44], all of which contained high densities of THli fibers (see Fig 3A_2_, 3B_2_ and 3B_3_). As in the TN, small neurons were frequently observed near branch points of the large oral veil nerve (LOVN), a division of the OVN containing sensory afferents (Fig 7B_1_).

The oral veil epithelium contained irregular distributions of THli structures, including elongated terminations situated in oral veil papillae (Fig 7B_2_). It was possible to resolve very small somata (<5 μm) giving rise to these terminations, which appeared as tightly stacked cilia-like formations arranged in parallel to one another that penetrated the epithelium (Fig 7B_3_). In other planes of focus, these cell bodies were observed to project axons into adjacent branches of the LOVN, which themselves were diffusely arranged in subepithelial layers of the oral veil. Individual papillae possessed as many as half a dozen cilia-like formations distributed in all three dimensions. Similar THli epithelial neurons were observed in the tentacle.

### Behavior

**Fig 8:**
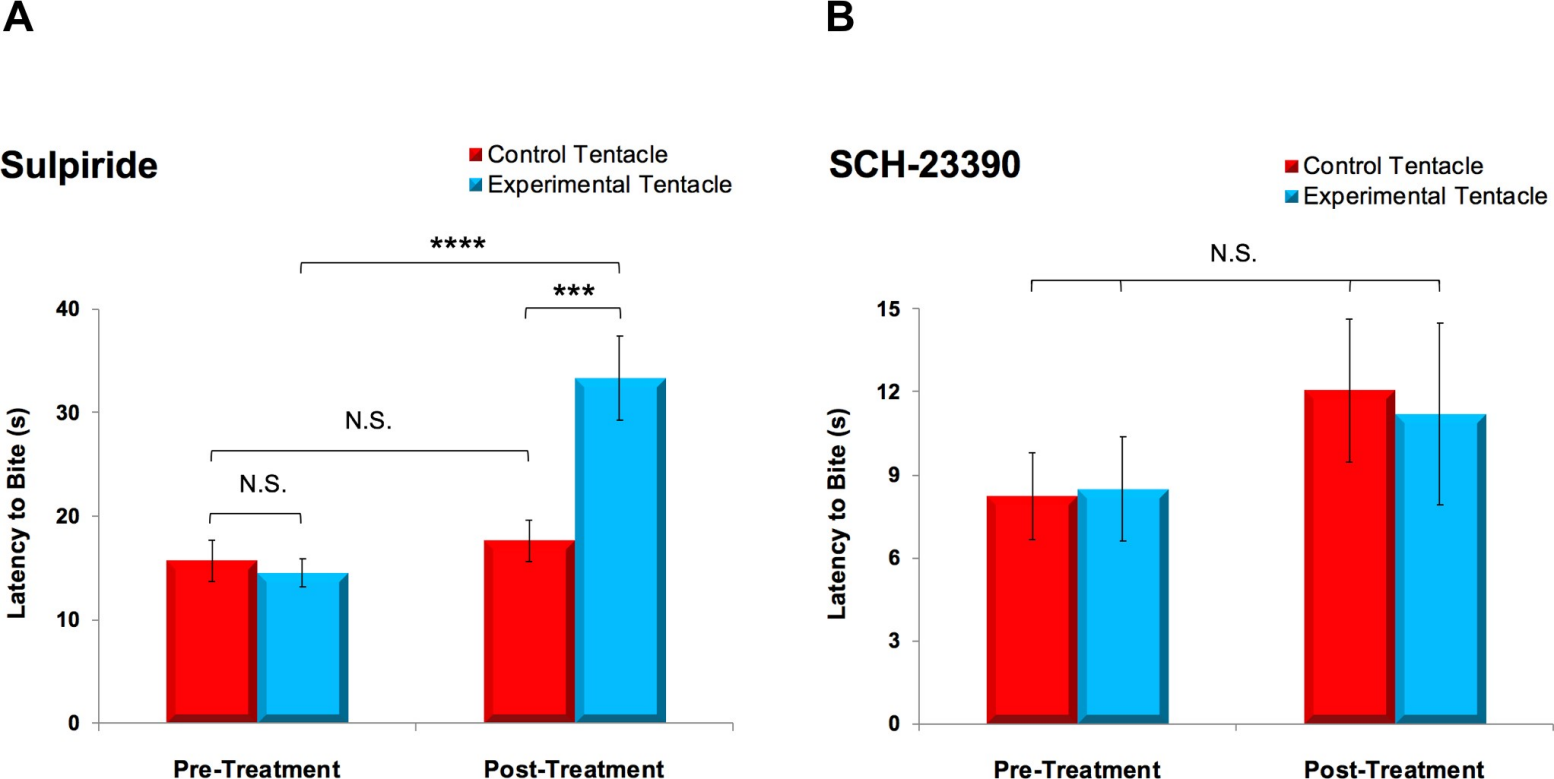
The D_2_/D_3_ dopamine antagonist sulpiride increased latency to bite in a food-localization task. (A) Unilateral application of sulpiride to the OVTC of *Pleurobranchaea* (N = 24) significantly increased the latency to bite at shrimp (two-way repeated measures ANOVA, *F*(1,23) = 13.28, *p* = 0.0014). Sulpiride increased latency to bite at stimuli placed on the experimental tentacle by 18.8 ± 3.1 s (mean ± SEM, *p* < 0.0001), with post-treatment latencies measured on the experimental tentacle 15.7 ± 3.1 s longer than those on the control tentacle (*p* = 0.0002). (B) Bite latencies did not significantly change on either side of the OVTC when food was presented to animals (N = 6) treated with the D_1_ antagonist SCH-23390 (two-way repeated measures ANOVA, *F*(1,5) = 1.21, *p* = 0.321). Individual bars depict means with SEMs. ****, *p* < 0.0001; ***, *p* < 0.001; N.S. = not significant.

To assess the effects of impeding DAergic transmission in *Pleurobranchaea*’s sensory periphery, we topically applied two different DA antagonist solutions to the OVTC of behaving animals and measured changes in the latency to bite in a food-seeking task. A two-way repeated measures ANOVA performed on log-transformed data revealed that the D_2_/D_3_ antagonist sulpiride significantly increased latency to bite at a food stimulus placed on the treated tentacle (N = 24), *F*(1,23) = 13.28, *p* = 0.0014 (Fig 8A). Simple main effects analysis disclosed that latencies measured at the experimental tentacle increased by 18.8 ± 3.1 s (mean ± SEM, *p* < 0.0001) when food was presented following treatment, with latencies to bite 15.7 ± 3.1 s (*p* = 0.0002) longer on the experimental tentacle relative to the control tentacle following sulpiride application. There was no significant difference between the latencies measured on the control tentacle before and after treatment (2.0 ± 3.1 s, *p* = 0.540), nor was there any difference between the latencies for the two tentacles during pre-treatment trials (1.1 ± 3.1 s, *p* = 1.000). In all cases, the impetus to feed was preserved following sulpiride treatment.

In contrast to the animals treated with sulpiride, specimens (N = 6) treated with the D_1_ antagonist SCH-23390 failed to exhibit significant changes in bite latency, as revealed by a two-way repeated measures ANOVA on log-transformed data, *F*(1,5) = 1.21, *p* = 0.321 (Fig 8B). Main effects analysis established that there were neither significant changes in latency following treatment (*p* = 0.680) nor between the experimental and control tentacles (*p* = 0.654).

After the stimulus was initially placed on a tentacle, specimens typically moved their heads to center their mouths around the shrimp, brushing increasingly more medial segments of their oral veils along the stimulus during the process. In addition to requiring greater time when food was presented to the tentacle treated with sulpiride, centering of the mouth around the stimulus became more erratic, with animals sometimes moving their mouths towards and away from the shrimp piece before biting commenced; such discrepancies were not observed following treatment with SCH-23390. In certain instances both before and after treatment with sulpiride, specimens extended their proboscises and even bit before their mouths were centered at the stimulus. Neither the mechanics of proboscis extension nor biting were noticeably affected by treatment with either pharmacological agent. A paired t-test on log-transformed measurements (N = 6) demonstrated that application of pure DMSO, used for the initial dissolving of sulpiride, to one side of the OVTC did not significantly change latency to bite when a food stimulus was presented to the treated tentacle (5.0 ± 3.0 s, *t*(5) = 1.643, *p* = 0.161); this affirmed that the effects observed were attributable to sulpiride.

### Electrophysiology

**Fig 9:**
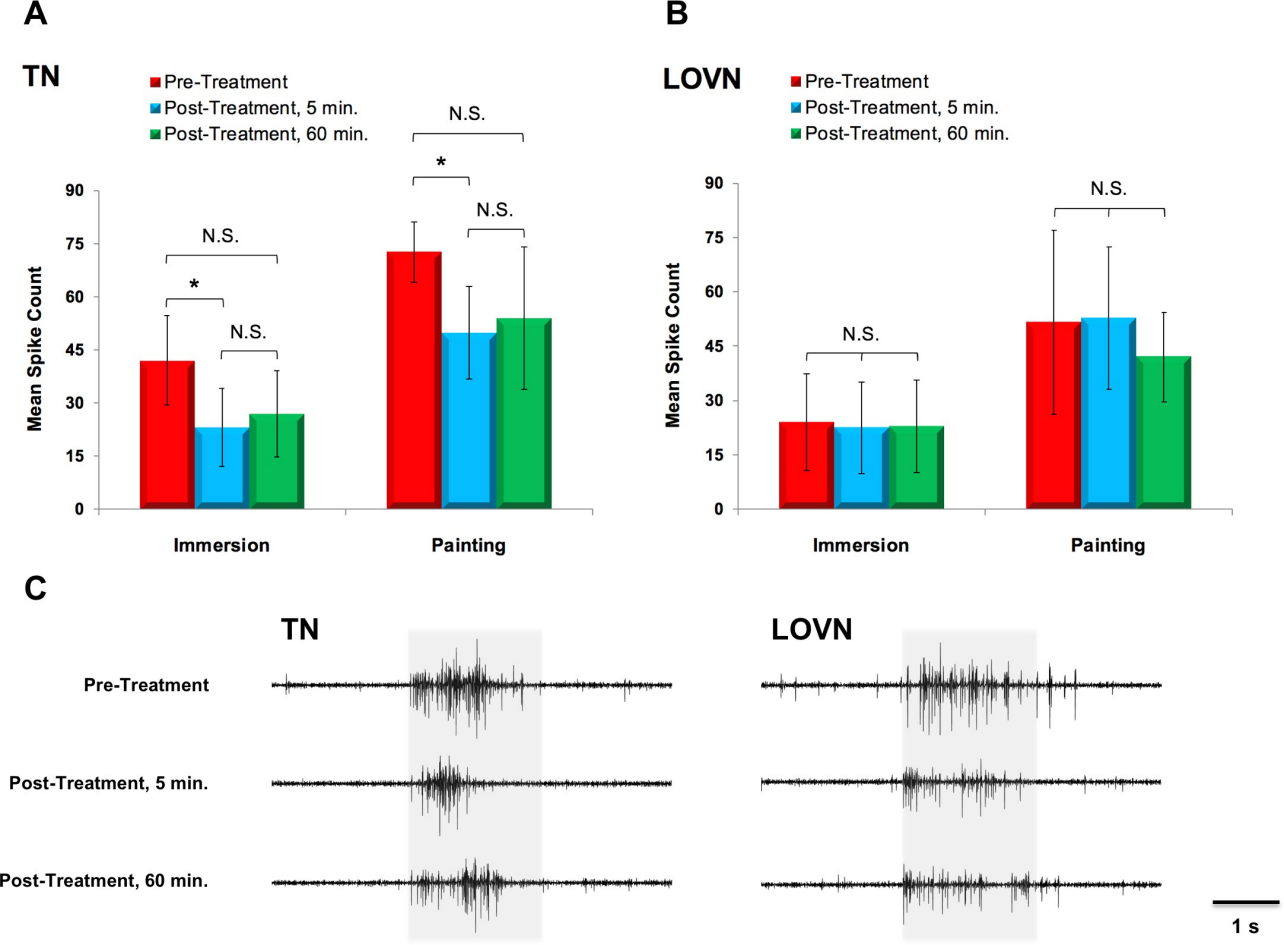
The D_2_/D_3_ dopaminergic antagonist sulpiride significantly reduced sensory responses to tactile stimuli measured in the tentacle nerve (TN). (A) Stimulus-evoked activity in the TN was attenuated by treating the OVTC with sulpiride, both through immersion (weighted-means one-way repeated measures ANOVA, *F*(2,18) = 5.75, *p* = 0.0117) and painting (*F*(2,9) = 4.98, *p* = 0.0350). When measured at 5 minutes following sulpiride treatment, TN activity had decreased by 18.81 ± 16.81 (mean ± SEM; Tukey’s test, *p* = 0.0130) in immersed preparations and by 22.85 ± 15.68 spikes (*p* = 0.0288) in preparations that had been painted. Evoked activity in the TN at 60 minutes following treatment was not significantly different than pre-treatment levels but exhibited no significant recovery relative to activity elicited through stimulation at 5 minutes post-treatment. (B) Evoked activity in the LOVN was not significantly changed following sulpiride treatment in either immersed (weighted-means one-way repeated measures ANOVA, *F*(2,18) = 0.07, *p* = 0.937) or painted preparations (*F*(2,6) = 0.74, *p* = 0.517). Individual bars depict weighted means with SEMs. *, *p* < 0.05; N.S. = not significant. TN: N = 11 for immersion, N = 7 for painting; LOVN, N = 11 for immersion, N = 5 for painting. (C) Sets of representative electrophysiological records obtained from a single preparation demonstrate responses in the TN and LOVN to tactile stimulation before, 5 minutes after, and 60 minutes after sulpiride treatment. Shading represents the 2 s over which tactile stimulation was applied.

As an extension of the behavioral observations, we applied sulpiride to the OVTC of deganglionated head preparations in two different manners to measure the effect of the reagent on stimulus-evoked responses in the TN and LOVN. Weighted-means one-way repeated measures ANOVAs demonstrated that sulpiride significantly reduced spiking responses to tactile stimuli in the TN, both when the reagent was applied through immersion of the preparation in sulpiride solution, *F*(2,18) = 5.75, *p* = 0.0117 (data log-transformed), or through painting the OVTC, *F*(2,9) = 4.98, *p* = 0.0350 (Fig 9A). Tukey’s tests revealed that relative to pre-treatment measurements, evoked nerve activity at 5 min. following sulpiride treatment had decreased significantly, by 18.81 ± 16.81 spikes when preparations were immersed (*p* = 0.0130) and 22.85 ± 15.68 spikes when they were painted (*p* = 0.0288). When measured at 60 min. following treatment, stimulus-evoked TN activity had increased, but not significantly relative to levels at 5 min. following sulpiride treatment (differences: immersion, 3.71 ± 16.39 spikes, *p* = 0.785; painting, 4.12 ± 23.99 spikes, *p* = 0.428); however, evoked activity at 60 minutes was not significantly different than pre-treatment levels (differences: immersion, 15.10 ± 17.54 spikes, *p* = 0.086; painting, 18.73 ± 21.77 spikes, *p* = 0.413).

Sulpiride did not exert a significant effect on stimulus-driven activity in the LOVN, regardless of how the reagent was applied (Fig 9B): in instances where preparations were immersed in a sulpiride bath, a weighted-means one-way repeated measures ANOVA on log-transformed data revealed insignificant changes in spike counts across the three testing epochs, *F*(2,18) = 0.07, *p* = 0.937, while the same analysis on data collected in experiments where the OVTC was painted similarly disclosed no significant effects, *F*(2,6) = 0.74, *p* = 0.517. Representative electrophysiological traces demonstrating the effects of sulpiride on stimulus-evoked responses in the TN and LOVN are shown in Fig 9C.

## Discussion

Dopamine localization in *Pleurobranchaea* is generally similar to that of other heterobranch gastropods. Seven genera, now including *Pleurobranchaea*, with *Aplysia*, *Biomphalaria*, *Helix*, *Helisoma*, *Lymnaea*, and *Phestilla*, possess substantially larger representations of THli in the PNS relative to the CNS (cf. [9,11,21,25,45,46]). An eighth, *Limax*, shows a similar pattern of catecholamine fluorescence (likely DA-based; [47]). Three more genera, *Acteon*, *Haminoea*, and *Archidoris*, also exhibit peripheral representation of THli in cephalic sensory structures, although without CNS comparison [24]. Thus, the character of prominent DA association with the PNS appears common across heterobranchs. Together with the findings that 1) impairing cephalic DAergic transmission delayed the initiation of biting at a food stimulus and 2) treating the cephalic sensory organs with a D_2_/D_3_ antagonist significantly diminished stimulus-evoked activity in the innervating tentacle nerve, we conclude that DA is a probable mediator and/or modulator of sensation in this species, and likely throughout the clade. The association of DA with sensory pathways in gastropods offers both interesting similarities and contrasts to DA localization and functions in vertebrates and arthropods, with potential relevance to the evolution of higher-order DAergic functions.

### Dopamine in the central nervous system

The observed localization of THli in the central nervous system of *Pleurobranchaea* agreed generally with that found in the two other opisthobranch molluscs available for comparison, with differences largely in numbers of immunoreactive cells in specific ganglia. Few deductions can be drawn regarding the significance of the variability in soma numbers; however, the conservation of the soma groups in the ganglia among the surveyed species is consistent with the conservation of basic neuronal circuitry in the broad diversification of the opisthobranchs. This is notable in the neural circuit that controls feeding, which has been intensively studied in multiple species, and is proposed to be controlled by a highly conserved central pattern generator network across opisthobranchs and pulmonates [48–50]. This hypothesis is supported by the high conservation of dopaminergic neuron numbers and locations in the buccal ganglia of the species studied. The marked conservation of DAergic neuron groups in the various ganglia across such diverse species is consistent with observations that neural circuitry is highly conserved in evolution relative to the marked changes in somatic morphology associated with new lifestyles and physiologies [51–53].

The central localization of THli neurons may reflect dopaminergic contributions to motor control in many cases. THli cells in the lateral buccal ganglion of *Pleurobranchaea* (Fig 5) appear similar to neurons in *Aplysia* (B65) and *Helisoma* (N1a) that are known to drive feeding motor programs [18–20]. The more medial bipolar THli cells are also putatively homologous to DAergic interneurons in *Aplysia* (B20) and *Helisoma* (N1b) that specify ingestive motor programs [16,48]. The buccal THli neurons in *Pleurobranchaea* and other gastropods may function similarly to promote and shape motor programs.

### Dopamine as a contributor to sensation in gastropods

Immunoreactivity in the cephalic PNS was observed in at least two layers of probable sensory neurons (Fig 7): 1) within the ciliated primary receptors of the OVTC epithelium and 2) monopolar interneurons with peripheral somata and axons in the TN and LOVN that project to the CNS. Together, these neurons are thought to participate in both sensory transduction and downstream integration [43]. Ciliated epithelial cells are associated with both mechanoreception [9] and chemoreception [54,55] in different systems, but their functions have not yet been rigorously tested. Sensory function is similarly inferred for the profusely labeled axons in the LOVN, a sensory nerve, and by analogy the labeled processes in the mixed sensory and motor TN, most of which likely originate in THli somata of the tentacle ganglia, are also likely sensory [44]. At least some of the immunoreactive fibers within the TN and LOVN are thought to stem from the abundance of small, tightly clustered neurons in close proximity to the central origins of these nerves (cf. [56–58]), although it is likely that the larger portion of stained fibers located in these sensory nerves derives from peripheral cell bodies, consistent with the conclusion of Croll et al. [59] for *Lymnaea*. Moreover, labeled small cell bodies at the branch junctions of the TN and LOVN could mediate peripheral interactions between sensory cells and/or local actions in the musculature, and actions in the CNS, as was suggested by Croll [9].

THli staining in the oral veil PNS of *Pleurobranchaea* agreed well with catecholamine-positive localization in other gastropods: THli somata were observed in the sensory epithelium, and extensive THli innervation was found throughout the sensory organs. Stained epithelial neurons in the oral veil (Fig 7B_2_ and 7B_3_) resembled THli neurons described in other genera, including *Aplysia*, *Phestilla*, *Acteon*, *Archidoris*, *Biomphalaria* and *Lymnaea*. These cells were in all cases bipolar and penetrated the epithelium with multiple cilia-like projections [9,11,24,25,46]. THli has been observed in putative sensory receptors of rhinophores, rhinophore ganglia, the mouth, foregut, non-cephalic body wall, and foot of other gastropods [24,46]. In *Aplysia*, dopaminergic afferents from anterior sensory receptors in the buccal cavity are proposed to mediate unconditioned stimuli in both classical and operant conditioning of feeding responses [3,60–64] (see also [36]).

The hypothesis that DAergic structures in the cephalic periphery of gastropods could serve sensory functions was supported by both behavioral and electrophysiological observations in this study. The increased latency-to-bite in a food-localization task indicates that topically applied sulpiride penetrated the sensory epithelium. We deduce that it proceeded to antagonize D_2_- and/or D_3_-like dopamine receptors on cells innervated by putatively DAergic primary receptors and possibly interneurons in the tentacle ganglion and OVTC subepithelium; it is also possible that some of the affected neurons were autoreceptive for DA. These conclusions follow from the observations that sulpiride treatment on one side of the OVTC in behaving animals 1) increased the latency to bite at an appetitive chemotactile stimulus applied to the treated tentacle and 2) effected no change in the latency to bite at a stimulus placed on the untreated tentacle. Furthermore, that sulpiride did not impact overall readiness-to-feed, as measured by each specimen’s feeding threshold, negates the possibility that the specimens’ delay in locating food stimuli was attributable to other potential effects of sulpiride on feeding behavior, such as decreasing appetite or feeding incentive. The fact that specimens were eventually able to detect the presence of and bite at a food stimulus indicates that any sulpiride-driven sensory attenuation underlying the delay to bite was incomplete. This observation was mirrored in the electrophysiological paradigm, in which sulpiride tended to diminish but not altogether abolish stimulus-evoked activity in the TN. Indeed, if taken together, the behavioral and electrophysiological results reported here suggest that the increased latency to bite when food was presented to the sulpiride-treated tentacle might correlate with the sulpiride-mediated reduction in the relaying of sensory information to the CNS: reduced or confounded afferent transmission from the lateral aspect of the OVTC, which is innervated by the TN, could conceivably impair food localization.

Several comments should be made relative to the electrophysiological results. First, the apparent discrepancy between the effects of sulpiride on the TN and LOVN may reflect the differing anatomies of these two nerves: unlike the TN, which receives input from a tentacle ganglion consisting of mechano- and chemoreceptive somata innervating the OVTC epithelium, the LOVN is not known to associate with any peripheral ganglia [43]. In light of the THli somata and neuropil identified in the tentacle ganglion, it is conceivable that neuronal populations in this ganglion projecting into the TN were influenced by sulpiride in a manner not affecting neurons projecting into the LOVN. Secondly, we surmise that the lack of significant recovery in the TN at 60 min. following sulpiride treatment owe to the drug possessing a longer half-life in molluscan tissue than originally anticipated. Although we have found that nominal physiology in the reduced preparation used in this study cannot be maintained for much longer than an hour, we predict that a more robust recovery from sulpiride treatment would be observed if the preparation could be maintained and tested several hours following treatment. Finally, we have previously noticed appreciable variability in recordings of nerve responses of the isolated OVTC from preparation to preparation that do not seem nearly so notable in semi-intact preparations, where the CNS remains attached; this same variability was observed in the present study. This is possibly due to loss of serotonergic output from the CNS as an arousal factor, in combination with loss of hydrostatic turgor in the dissection [65]. In light of this, electrophysiology employing the same pharmacological manipulation in preparations with intact CNS or in whole-animal preparations is warranted and represents a future direction of this work.

The significant but partial effects exerted by sulpiride in both behavioral and electrophysiological experiments invite speculation as to the exact role played by DA vis-à-vis sensation in *Pleurobranchaea*. If DA acts by directly mediating synaptic transmission along afferent pathways to the CNS, there may be other neurotransmitters acting in parallel to relay sensory information from the periphery. Alternatively, the extent to which DA receptors are antagonized may be limited by sulpiride’s kinetics or diffusive properties in molluscan tissue. DA could also function in a modulatory manner within the sensory periphery, perhaps analogous to the role it and other neuromodulators serve in setting the transduction threshold for tail sensory neurons in *Aplysia* [66]. Elucidating the precise neurochemistry of DA relative to peripheral sensory processing in gastropods ultimately requires a more thorough anatomical characterization of peripheral sensory infrastructure in these animals than is presently available.

### Evolution of the dopaminergic system

Dopamine participates in diverse functions across the phyla, ranging from the integration of sensory input in associative learning to signaling reward and its prediction [1–3,67–70]. However, evidence is presently scarce for the participation of DA in sensation itself at levels of transduction and peripheral integration. An exception is the demonstration of DAergic mechanosensory neurons in the nematode *Caenorhabditis elegans*, some of which detect edible bacteria [71–74]. Like the THli putative primary receptors in *Pleurobranchaea* and other gastropods, these cells in *C*. *elegans* project ciliated endings into the epithelium [2]. Although histologically a component of the vertebrate CNS, the olfactory bulb expresses large population of DAergic juxtaglomerular neurons [75]; it is conceivable, given the single intervening synapse between the olfactory epithelium and bulb, that a structure analogous to the latter was located peripherally in evolutionary antecedents.

DA has common functions in action selection, attention, and reward in deuterostomes and protostomes, which suggests analogous roles in the ancestral bilaterian nervous systems [33]. Thus, it is notable that in *Pleurobranchaea* and other gastropods, most DAergic neurons are located in the PNS and associated with primary appetitive sensory pathways. Accordingly, we speculate that the peripheral dopaminergic system of gastropods may act within the peripheral sensory network to integrate incentive and reward, and thereby resembles DA-dependent processing in the vertebrate mesolimbic system [67]. Previously, it was shown that the feeding motor network of *Pleurobranchaea* expresses appetitive state, the moment-to-moment integration of sensation, memory, and satiation, in the excitation state of the network [37,76,77]. Moreover, the feeding network excitation state directs decisions for approach or avoidance of appetitive stimuli through corollary outputs that alter the turn motor network’s response from avoidance to approach [76,77]. In this respect, it was proposed [78] that the feeding network of *Pleurobranchaea* embodies functions of the vertebrate hypothalamus in determining stimulus incentivization via motivational state [79] and of the basal ganglia in determining behavioral choice [80]. On this point, the correspondence of molluscan and vertebrate nervous system organizations may in fact constitute functional homologies in the organization of sensory integration and motor control. The precise role of dopamine in valuation, sensory gating, and behavioral choice may be quite accessible to investigation in *Pleurobranchaea* and other gastropod model systems.

## Supporting information

S1 Dataset Behavioral dataset. (XLSX)

S2 Dataset Electrophysiological dataset. (XLSX)

## References

[1] Montague PR, Dayan P, Sejnowski TJ. A framework for mesencephalic dopamine systems based on predictive Hebbian learning. J Neurosci. 1996;16:1936–47. 8774460 10.1523/JNEUROSCI.16-05-01936.1996 PMC6578666

[2] Sawin ER, Ranganathan R, Horvitz HR.*C*. *elegans* locomotory rate is modulated by the environment through a dopaminergic pathway and by experience through a serotonergic pathway. Neuron. 2000;26:619–31. 10896158 10.1016/s0896-6273(00)81199-x

[3] Brembs B, Lorenzetti FD, Reyes FD, Baxter DA, Byrne JH. Operant reward learning in *Aplysia*: Neuronal correlates and mechanisms. Science. 2002;296:1706–9. 10.1126/science.1069434 12040200

[4] Schultz W. Dopamine signals for reward value and risk: basic and recent data. Behav Brain Funct. 2010;6:24 10.1186/1744-9081-6-24 20416052 PMC2876988

[5] Schultz W. Updating dopamine reward signals. Curr Opin Neurobiol. 2013;23:229–38. 10.1016/j.conb.2012.11.012 23267662 PMC3866681

[6] Strausfeld NJ, Hirth F. Deep homology of arthropod central complex and vertebrate basal ganglia. Science. 2013;340:157–61. 10.1126/science.1231828 23580521

[7] Elekes K, Kemenes G, Hiripi L, Geffard M, Benjamin PR. Dopamine-immunoreactive neurones in the central nervous system of the pond snail *Lymnaea stagnalis*. J Comp Neurol. 1991;307:214–24. 10.1002/cne.903070205 1713231

[8] Hernádi L, Elekes K. Topographic organization of serotonergic and dopaminergic neurons in the cerebral ganglia and their peripheral projection patterns in the head areas of the snail *Helix pomatia*. J Comp Neurol. 1999;411:274–87. 10404253 10.1002/(sici)1096-9861(19990823)411:2<274::aid-cne8>3.0.co;2-9

[9] Croll RP. Catecholamine-containing cells in the central nervous system and periphery of *Aplysia californica*. J Comp Neurol. 2001;441:91–105. 11745637 10.1002/cne.1399

[10] Croll RP, Boudko DY, Hadfield MG. Histochemical survey of transmitters in the central ganglia of the gastropod mollusc *Phestilla sibogae*. Cell Tissue Res. 2001;305:417–32. 11572095 10.1007/s004410100394

[11] Vallejo D, Habib MR, Delgado N, Vaasjo LO, Croll RP, Miller MW. Localization of tyrosine hydroxylase-like immunoreactivity in the nervous systems of *Biomphalaria glabrata* and *Biomphalaria alexandrina*, intermediate hosts for schistosomiasis. J Comp Neurol. 2014;522:2532–52. 10.1002/cne.23548 24477836 PMC4043854

[12] Carpenter D, Breese G, Schanberg S, Kopin I. Serotonin and dopamine: distribution and accumulation in *Aplysia* nervous and non-nervous tissues. Int J Neurosci. 1971;2:49–56. 5161303 10.3109/00207457109146992

[13] Trimble DL, Barker DL. Activation by dopamine of patterned motor output from the buccal ganglia of *Helisoma trivolvis*. J Neurobiol. 1984;15:37–48. 10.1002/neu.480150105 6321653

[14] Wieland SJ, Gelperin A. Dopamine elicits feeding motor program in *Limax maximus*. J Neurosci. 1983;3:1735–45. 6886743 10.1523/JNEUROSCI.03-09-01735.1983 PMC6564474

[15] Kyriakides MA, Mccrohan CR. Effect of putative neuromodulators on rhythmic buccal motor output in *Lymnaea stagnalis*. J Neurobiol. 1989;20:635–50. 10.1002/neu.480200704 2794997

[16] Teyke T, Rosen SC, Weiss KR, Kupfermann I. Dopaminergic neuron B20 generates rhythmic neuronal activity in the feeding motor circuitry of *Aplysia*. Brain Res. 1993;630:226–37. 8118689 10.1016/0006-8993(93)90661-6

[17] Kabotyanski EA, Baxter DA, Cushman SJ, Byrne JH. Modulation of fictive feeding by dopamine and serotonin in *Aplysia*. J Neurophysiol. 2000;83:374–92. 10.1152/jn.2000.83.1.374 10634881

[18] Díaz-Ríos M, Miller MW. Rapid dopaminergic signaling by interneurons that contain markers for catecholamines and GABA in the feeding circuitry of *Aplysia*. J Neurophysiol. 2005;93:2142–56. 10.1152/jn.00003.2004 15537820

[19] Kabotyanski EA, Baxter DA, Byrne JH. Identification and characterization of catecholaminergic neuron B65, which initiates and modifies patterned activity in the buccal ganglia of *Aplysia*. J Neurophysiol. 1998;79:605–21. 10.1152/jn.1998.79.2.605 9463425

[20] Quinlan EM, Arnett BC, Murphy AD. Feeding stimulants activate an identified dopaminergic interneuron that induces the feeding motor program in *Helisoma*. J Neurophysiol. 1997;78:812–24. 10.1152/jn.1997.78.2.812 9307115

[21] Croll RP, Chiasson BJ. Distribution of catecholamines and of immunoreactivity to substances like vertebrate enzymes for the synthesis of catecholamines within the central nervous system of the snail, *Lymnaea stagnalis*. Brain Res. 1990;525:101–14. 1978788 10.1016/0006-8993(90)91325-b

[22] Syed NI, Roger I, Ridgway RL, Bauce LG, Lukowiak K, Bulloch AGM. Identification, characterization, and *in vitro* reconstruction of an interneuronal network of the snail, *Helisoma trivolvis*. J Exp Biol. 1993;174:19–44. 8440965 10.1242/jeb.174.1.19

[23] Salimova NB, Sakharov DA, Milosevic I, Rakic L. Catecholamine-containing neurons in the peripheral nervous system of *Aplysia*. Acta Biol Hung. 1987;38:203–12. 3454082

[24] Faller S, Stauback S, Klussman-Kolb A. Comparative immunohistochemistry of the cephalic sensory organs in Opisthobranchia (Mollusca, Gastropoda). Zoomorphology. 2008:127:227–39.

[25] Wyeth RC, Croll RP. Peripheral sensory cells in the cephalic sensory organs of *Lymnaea stagnalis*. J Comp Neurol. 2011;519:1894–1913. 10.1002/cne.22607 21452209

[26] Carrigan ID, Croll RP, Wyeth RC. Morphology, innervation, and peripheral sensory cells of the siphon of *Aplysia californica*. J Comp Neurol. 2015;523:2409–25. 10.1002/cne.23795 25921857

[27] Noboa V, Gillette R. Selective prey avoidance learning in the predatory sea slug *Pleurobranchaea californica*. J Exp Biol. 2013;216:3231–6. 10.1242/jeb.079384 23661778

[28] Yafremava LS, Gillette R. Putative lateral inhibition in sensory processing for directional turns. J Neurophysiol. 2011;105:2885–90. 10.1152/jn.00124.2011 21490281 PMC3118739

[29] Magoski NS, Bauce LG, Syed NI, Bulloch AGM. Dopaminergic transmission between identified neurons from the mollusk, *Lymnaea stagnalis*. J europhysiol. 1995;74:1287–1300. 10.1152/jn.1995.74.3.1287 7500151

[30] Serrano GE, Miller MW. Conditional rhythmicity and synchrony in a bilateral pair of bursting motor neurons in *Aplysia*. J Neurophysiol. 2006;96:2057–71. 10.1152/jn.00282.2006 16738215

[31] Narusuye K, Nagahama T. Cerebral CBM1 Neuron Contributes to Synaptic Modulation Appearing During Rejection of Seaweed in *Aplysia kurodai*. J Neurophysiol. 2002;88:2778–95. 10.1152/jn.00757.2001 12424312

[32] Mukai ST, Kiehn L, Saleuddin ASM. Dopamine stimulates snail albumen gland glycoprotein secretion through the activation of a D1-like receptor. J Exp Bio. 2004;207:2507–18. 15184522 10.1242/jeb.01052

[33] Barron AB, Søvik E, Cornish JL. The roles of dopamine and related compounds in reward-seeking behavior across animal phyla. Front Behav Neurosci. 2010;4:163 10.3389/fnbeh.2010.00163 21048897 PMC2967375

[34] Waddell S. Reinforcement signalling in *Drosophila*; dopamine does it all after all. Curr Opin Neurobiol. 2013;23:324–9. 10.1016/j.conb.2013.01.005 23391527 PMC3887340

[35] Díaz-Ríos M, Oyola E, Miller MW. Colocalization of gamma-aminobutyric acid-like immunoreactivity and catecholamines in the feeding network of *Aplysia californica*. J Comp Neurol. 2002;445:29–46. 11891652 10.1002/cne.10152

[36] Martínez-Rubio C, Serrano GE, Miller MW. Localization of biogenic amines in the foregut of *Aplysia californica*: catecholaminergic and serotonergic innervation. J Comp Neurol. 2009;514:329–42. 10.1002/cne.21991 19330814 PMC4023389

[37] Gillette R, Huang RC, Hatcher N, Moroz LL. Cost-benefit analysis in feeding behavior of a predatory snail by integration of hunger, taste, and pain. Proc Natl Acad Sci USA. 2000;97:3585–90. 10737805 10.1073/pnas.97.7.3585 PMC16283

[38] Sudlow LC, Jing J, Moroz LL, Gillette R. Serotonin immunoreactivity in the central nervous system of the marine molluscs *Pleurobranchaea californica* and *Tritonia diomedea*. J Comp Neurol. 1998;395:466–80. 9619500

[39] Jing J, Gillette R. Neuronal elements that mediate escape swimming and suppress feeding behavior in the predatory sea slug *Pleurobranchaea*. J Neurophysiol. 1995;74:1900–10. 10.1152/jn.1995.74.5.1900 8592183

[40] Bicker G, Davis WJ, Matera EM, Kovac MP, StormoGipson DJ. Chemoreception and mechanoreception in the gastropod mollusc *Pleurobranchaea californica*. I. Extracellular analysis of afferent pathways. J Comp Physiol. 1982;149:221–34.

[41] Rosen SC, Teyke T, Cohen JL, Miller MW, Weiss KR, Kupfermann I. Identification and characterization of cerebral-to-buccal interneurons implicated in the control of motor programs associated with feeding in *Aplysia*. J Neurosci. 1991;11:3630–55. 1941100 10.1523/JNEUROSCI.11-11-03630.1991 PMC6575545

[42] Cohan CS, Mpitsos GJ. Selective recruitment of interganglionic interneurones during different motor patterns in *Pleurobranchaea*. J Exp Biol. 1983;102:43–57. 6833946 10.1242/jeb.102.1.43

[43] Bicker G, Davis WJ, Matera EM. Chemoreception and mechanoreception in the gastropod mollusc *Pleurobranchaea californica*. II. Neuroanatomical and intracellular analysis of afferent pathways. J Comp Physiol. 1982;149:235–50.

[44] Lee RM, Liegeois RJ. Motor and sensory mechanisms of feeding in *Pleurobranchaea*. J Neurobiol. 1974;5:545–64. 10.1002/neu.480050606 4436674

[45] Kiehn L, Saleuddin S, Lange A. Dopaminergic neurons in the brain and dopaminergic innervation of the albumen gland in mated and virgin *Helisoma duryi* (Mollusca: Pulmonata). BMC Physiology 2001;1:9 11513757 10.1186/1472-6793-1-9 PMC37538

[46] Croll RP, Boudko DY, Pires A, Hadfield MG. Transmitter contents of cells and fibers in the cephalic sensory organs of the gastropod mollusc *Phestilla sibogae*. Cell Tissue Res. 2003;314:437–48. 10.1007/s00441-003-0778-1 14598161

[47] Osborne NN, Cottrell GA. Distribution of biogenic amines in the slug, *Limax maximus*. Z Zellforsch Mikrosk Anat. 1971;112:15–30. 5544256 10.1007/BF00665618

[48] Murphy AD. The neuronal basis of feeding in the snail, *Helisoma*, with comparisons to selected gastropods. Prog Neurobiol. 2001;63:383–408. 11163684 10.1016/s0301-0082(00)00049-6

[49] Elliott CJ, Susswein AJ. Comparative neuroethology of feeding control in molluscs. J Exp Bio. 2002;205:877–96. 11916985 10.1242/jeb.205.7.877

[50] Wentzell MM, Martínez-Rubio C, Miller MW, Murphy AD. Comparative neurobiology of feeding in the opisthobranch sea slug, *Aplysia*, and the pulmonate snail, *Helisoma*: evolutionary considerations. Brain Behav Evol. 2009;74:219–30. 10.1159/000258668 20029185 PMC2855281

[51] Dickinson PS. Homologous neurons control movements of diverse gill types in nudibranch molluscs. J Comp Physiol. 1979;131:277–83.

[52] Arbas EA, Meinertzhagen IA, Shaw SR. Evolution in nervous systems. Annu Rev Neurosci. 1991;14:9–38. 10.1146/annurev.ne.14.030191.000301 2031578

[53] Jing J, Gillette R, Weiss KR. Evolving Concepts of Arousal: Insights from Simple Model Systems. Rev Neurosci. 2009;20:405–27. 20397622 10.1515/revneuro.2009.20.5-6.405

[54] Matera EM, Davis WJ. Paddle cilia (discocilia) in chemosensitive structures of the gastropod mollusk *Pleurobranchaea californica*. Cell Tissue Res. 1982;222:25–40. 7060096 10.1007/BF00218286

[55] Emery DG. Fine structure of olfactory epithelia of gastropod molluscs. Microsc Res Tech. 1992;22:307–24. 10.1002/jemt.1070220402 1392062

[56] Audesirk G, Audesirk T. Complex Mechanoreceptors in *Tritonia diomedia*. J Comp Physiol. 1980;141:101–9.

[57] Nelson GM, Audesirk, TE. Identification of central neurons innervating peripheral chemoreceptive structures in *Lymnaea stagnalis*. Comp Biochem Physiol A Comp Physiol. 1986;83:113–20.

[58] Kemenes G. Processing of mechano- and chemosensory information in the lip nerve and cerebral ganglia of the snail *Helix pomatia* L. Neurosci Behav Physiol. 1994;24:77–87. 8208386 10.1007/BF02355656

[59] Croll RP, Voronezhskaya EE, Hiripi L, Elekes K. Development of catecholaminergic neurons in the pond snail, *Lymnaea stagnalis*: II. Postembryonic development of central and peripheral cells. J Comp Neurol. 1999;404:297–309. 9952349 10.1002/(sici)1096-9861(19990215)404:3<297::aid-cne2>3.0.co;2-i

[60] Nargeot R, Baxter DA, Byrne JH. Contingent-dependent enhancement of rhythmic motor patterns: an *in vitro* analog of operant conditioning. J Neuroscience 1997;17:8093–8105. 10.1523/JNEUROSCI.17-21-08093.1997 PMC6573721 9334385

[61] Nargeot R, Baxter DA, Patterson GW, Byrne JH. Dopaminergic synapses mediate neuronal changes in an analogue of operant conditioning. J Neurophysiol. 1999;81:1983–87. 10.1152/jn.1999.81.4.1983 10200235

[62] Lechner HA, Baxter DA, Byrne JH. Classical conditioning of feeding in *Aplysia*: II. Neurophysiological correlates. J Neurosci. 2000;20:3377–86. 10777800 10.1523/JNEUROSCI.20-09-03377.2000 PMC6773143

[63] Mozzachiodi R, Lechner HA, Baxter DA, Byrne JH. In vitro analog of classical conditioning of feeding behavior in *Aplysia*. Learning and Memory. 2003;10:478–94. 10.1101/lm.65303 14657259 PMC305463

[64] Baxter DA, Byrne JH. Feeding behavior of *Aplysia*: a model system for comparing cellular mechanisms of classical and operant conditioning. Learn Mem. 2006;13:669–80. 10.1101/lm.339206 17142299

[65] Moroz LL, Sudlow LC, Jing J, Gillette R. Serotonin immunoreactivity in peripheral tissues of the opisthobranch molluscs *Pleurobranchaea californica* and *Tritonia diomedea*. J Comp Neurol. 1997;382:176–88. 9183687 10.1002/(sici)1096-9861(19970602)382:2<176::aid-cne3>3.0.co;2-0

[66] Billy AJ, Walters ET. Modulation of mechanosensory threshold in *Aplysia* by serotonin, small cardioactive peptideB (SCPB), FMRFamide, acetylcholine, and dopamine. Neurosci Lett. 1989;150:200–4. 10.1016/0304-3940(89)90037-2 2577223

[67] O’Connell LA, Hofmann HA. The vertebrate mesolimbic reward system and social behavior network: A comparative synthesis. J Comp Neurol. 2011;519:3599–3639. 10.1002/cne.22735 21800319

[68] Schwaerzel M, Monastirioti M, Scholz H, Friggi-Grelin F, Birman S, Heisenberg M. Dopamine and octopamine differentiate between aversive and appetitive olfactory memories in *Drosophila*. J Neurosci. 2003;23:10495–502. 14627633 10.1523/JNEUROSCI.23-33-10495.2003 PMC6740930

[69] Wise RA. Dopamine, learning and motivation. Nat Rev Neurosci. 2004;5:483–94. 10.1038/nrn1406 15152198

[70] Schultz W. Behavioral dopamine signals. Trends Neurosci. 2007;3:203–10. 10.1016/j.tins.2007.03.007 17400301

[71] Sulston J, Dew M, Brenner S. Dopaminergic neurons in the nematode *Caenorhabditis elegans*. J Comp Neurol. 1975;163:215–26. 10.1002/cne.901630207 240872

[72] Hills T, Brockie PJ, Maricq AV. Dopamine and glutamate control area-restricted search behavior in *Caenorhabditis elegans*. J Neurosci. 2004;24:1217–25. 10.1523/JNEUROSCI.1569-03.2004 14762140 PMC6793574

[73] Sanyal S, Wintle RF, Kindt KS, Nuttley WM, Arvan R, Fitzmaurice P, et al Dopamine modulates the plasticity of mechanosensory responses in *Caenorhabditis elegans*. EMBO J. 2004;23:473–82. 10.1038/sj.emboj.7600057 14739932 PMC1271763

[74] Kindt KS, Quast KB, Giles AC, De S, Hendrey D, Nicastro I, et al Dopamine mediates context dependent modulation of sensory plasticity in *C*. *elegans*. Neuron. 2007;55:662–76. 10.1016/j.neuron.2007.07.023 17698017

[75] Baker H, Kawano T, Margolis FL, Joh TH. Transneuronal regulation of tyrosine hydroxylase expression in olfactory bulb of mouse and rat. J Neurosci. 1983;3:69–78. 6130133 10.1523/JNEUROSCI.03-01-00069.1983 PMC6564580

[76] Hirayama K, Gillette R. A neuronal network switch for approach/avoidance toggled by appetitive state. Curr Biol. 2012;22:118–23. 10.1016/j.cub.2011.10.055 22197246 PMC3267890

[77] Hirayama K, Moroz LL, Hatcher NG, Gillette R. Neuromodulatory control of a goal- directed decision. PLoS ONE. 2014;9(7):e102240 10.1371/journal.pone.0102240 25048964 PMC4105495

[78] Gillette R, Brown JW. *Pleurobranchaea californica* as a reference species signpost in evolution of complex nervous systems and behavior. Interg Comp Biol. 2015;55:1058–69. 10.1093/icb/icv081 PMC4836448 26163678

[79] Martin JR. Motivated behaviors elicited from hypothalamus, midbrain, and pons of the guinea pig (*Cavia porcellus*). J Comp Physiol Psychol. 1976;90:1011–34. 1033210 10.1037/h0078658

[80] Bogacz R, Gurney K. The basal ganglia and cortex implement optimal decision making between alternative actions. Neural Comput. 2007;19:442–77. 10.1162/neco.2007.19.2.442 17206871

